# New Recombinant Antimicrobial Peptides Confer Resistance to Fungal Pathogens in Tobacco Plants

**DOI:** 10.3389/fpls.2020.01236

**Published:** 2020-08-13

**Authors:** Mitra Khademi, Marzieh Varasteh-Shams, Farhad Nazarian-Firouzabadi, Ahmad Ismaili

**Affiliations:** Department of Agronomy and Plant Breeding, Faculty of Agriculture, Lorestan University, Khorramabad, Iran

**Keywords:** antifungal, chitin-binding domain, effector protein, expression, genetic engineering, transgenic plant

## Abstract

Antimicrobial peptides have been long known to confer resistance to plant pathogens. In this study, new recombinant peptides constructed from a dermaseptin B1 (DrsB1) peptide fused to a chitin-binding domain (CBD) from Avr4 protein, were used for *Agrobacterium tumefaciens*-mediated transformation of tobacco plants. Polymerase chain reaction (PCR), semi‐quantitative RT‐PCR, and western blotting analysis demonstrated the incorporation and expression of transgenes in tobacco genome and transgenic plants, respectively. *In vitro* experiments with recombinant peptides extracted from transgenic plants demonstrated a significant (P<0.01) inhibitory effect on the growth and development of plant pathogens. The DrsB1-CBD recombinant peptide had the highest antifungal activity against fungal pathogens. The expression of the recombinant peptides greatly protected transgenic plants from *Alternaria alternata, Alternaria solani, Fusarium oxysporum*, and *Fusarium solani* fungi, in comparison to *Pythium* sp. and *Pythium aphanidermatum*. Expression of new recombinant peptides resulted in a delay in the colonization of fungi and appearance of fungal disease symptoms from 6 days to more than 7 weeks. Scanning electron microscopy images revealed that the structure of the fungal mycelia appeared segmented, cling together, and crushed following the antimicrobial activity of the recombinant peptides. Greenhouse bioassay analysis showed that transgenic plants were more resistant to *Fusarium* and *Pythium* infections as compared with the control plants. Due to the high antimicrobial activity of the recombinant peptides against plant pathogens and novelty of recombinant peptides, this report shows the feasibility of this approach to generate disease resistance transgenic plants.

## Introduction

Global food security is continuously threatened by many growing risk factors, including rapid world population growth, and devastating plant parasites. It is estimated that biotic stresses reduce the world’s harvest and post-harvest up to 30% (Oerke, 2006; Flood, 2010). Although, biotic stress management has played a pivotal role in increasing food production in the last 50 years, plant pathogens still claim more than 10 and 6% of the global harvest and post-harvest, respectively (Strange and Scott, 2005; Oerke, 2006; Chakraborty and Newton, 2011).

Despite application of hazardous chemicals to prevent the colonization and growth of plant pathogens (Hahn, 2014; Drenth and Guest, 2016), the appearance of resistant isolates of devastating pathogens, especially fungi and oomycetes challenge the use of fungicides (Nicot et al., 2016). For instance, it has recently been documented that species belonging to *Alternaria* and *Phoma* have developed resistance against QoI or strobilurins and benzimidazole group of fungicides, respectively (Van de Graaf et al., 2003; Karaoglanidis et al., 2011; Kim et al., 2017), suggesting either new classes of fungicides must be designed or different approaches should be taken to overcome emerging plant pathogens.

Microbe or pathogen-associated molecular patterns (MAMPs/PAMPs) induce the immune responses in plants (Zipfel and Oldroyd, 2017; Saijo et al., 2018). In turn, microbial effectors target MAMP-triggered immunity in plants, mostly by interacting with the MAMP components leading to suppression of plant immunity responses (Ridout et al., 2006; Nürnberger and Kemmerling, 2009). Chitin, a long-chain polymer of N-acetylglucosamine, plays a crucial structural role in fungal cell walls and it is not produced by plant cells (Wan et al., 2008). Plant cells perceive chitin and chitooligosaccharides released from fungal invaders and trigger signaling *via* MAMP cascade initiating defense signaling through plasma membrane receptors (Kaku et al., 2006; Wan et al., 2008). In return, to hide a successful invasion, pathogens inhibit the recognition of chitin molecules by the host plant cells either by scavenging chitin or chitooligosaccharides molecules (De Jonge et al., 2010; Mentlak et al., 2012) or masking own cell wall chitin. For instance, *Cladosporium fulvum* Avr4 effector proteins bind to chitin molecules, hence, preventing host chitinases from degradation activity (Van den Burg et al., 2006), suggesting that MAMP-triggered immunity is a critical barrier to overcome plant pathogens damage to plants.

To target fungal cell walls, plants have acquired chitinases, enzymes of the glycoside hydrolase (GH) family that destroy chitin by hydrolyzing β-1,4 glycosidic bonds. Structurally, most known chitinases consist of two separate parts: a catalytic domain and a chitin-binding domain (CBD). It has been documented that the CBD helps the enzyme to attach to fungal cell wall chitin, thereby increasing the lytic activity of the catalytic domain (Iseli et al., 1993; Huang et al., 1997; Taira et al., 2002; Yan et al., 2008). Other than chitinases, CBDs are present in other protein families such as fungal effector proteins (Van den Burg et al., 2004) which play a major role as virulence factor (Van Esse et al., 2007).

Antimicrobial peptides (AMPs), short and conserved small proteins with less than 50 amino acids, are produced by a wide range of living organisms from bacteria to animals as a part of their innate defense mechanism (Bechinger and Gorr, 2017). The widespread distribution of AMPs among living organisms suggests their fundamental roles in the successful evolution of multicellular organisms (Yeaman and Yount, 2003; Mahlapuu et al., 2016). In addition to the naturally-occurring AMPs, considerable number of diverse group of recombinant AMPs have been designed to achieve AMPs higher stability, specificity (Ferre et al., 2006; Marcos et al., 2008; Badosa et al., 2013) and to enhance pathogen resistance in plants (Coca et al., 2006; Jan et al., 2010; Rivero et al., 2012). Recently, different antimicrobial peptides have been fused each other or functional proteins to provide bifunctional properties leading to a higher antibacterial activity than that of each AMP alone. For instance, cecropin A-Mag II hybrid antimicrobial peptide, exhibits much higher antibacterial activity than that of cecropin A or Mag II (Jin et al., 2006; Wang et al., 2012). Recent studies have shown that recombinant peptides can be used to improve disease resistance to different pathogens (Badrhadad et al., 2018; Khademi et al., 2019; Shams et al., 2019), opening a new path to engineer AMPs by fussing them to fusion partners.

Dermaseptins, cationic AMPs of 28 to 34 amino acids, are mainly isolated from frogs of the *Phyllomedusa* genus supplies both antibacterial and antifungal protection to a broad range of plant pathogens (Misra and Kay, 2004; Rivero et al., 2012). Dermaseptin B1 (DrsB1) is a 31 amino acids long AMP with an alpha-helix structure and it is one of the strongest AMPs known among all AMPs (Yevtushenko and Misra, 2007). In spite of the broad spectrum of antimicrobial activity against various pathogens, DrsB1 shows no toxic effects on plant and mammalian cells (Mor and Nicolas, 1994; Strahilevitz et al., 1994; Osusky et al., 2005), making it a good candidate for generating transgenic plants resistance to plant pathogens.

In our previous studies we had successfully shown that fusion of different CBDs to antimicrobial peptides significantly enhanced resistance of transgenic plants against bacterial and fungal pathogens *in vitro* (Khademi et al., 2019; Shams et al., 2019) and *in vivo* (Badrhadad et al., 2018). Here we report generating transgenic tobacco plants expressing different DrsB1 peptide resistance to plant pathogens. In the future, a thorough understanding of the mechanism of action of recombinant peptides will open up new opportunities for the development of more active and functional recombinant peptides.

## Materials and Methods

### Construction of Expression Vectors

**Figure 1:**
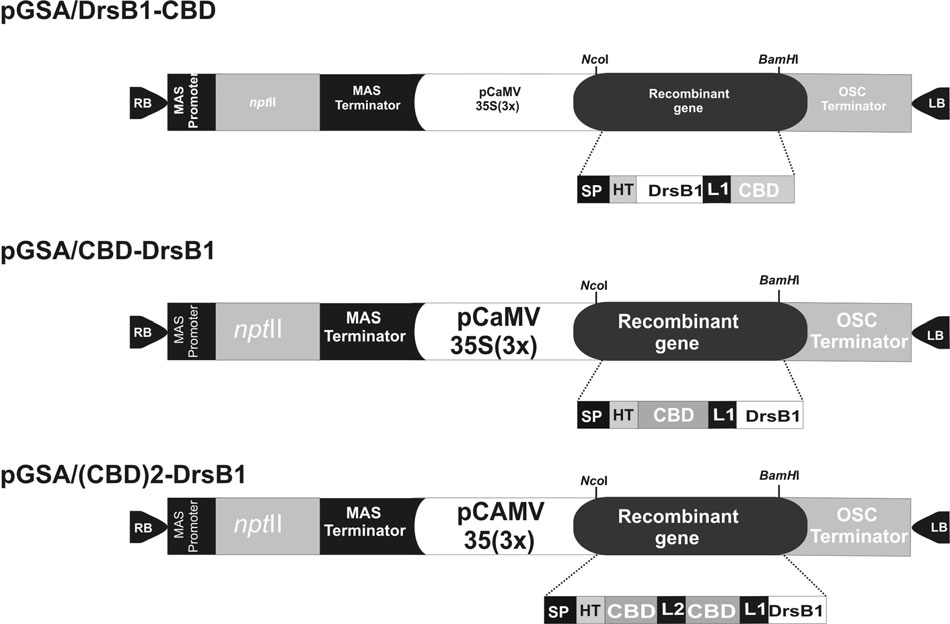
Schematic representation of the three genetic constructs used for Agrobacterium-mediated transformation. MAS, mannopine synthase; npt II: neomycin phosphotransferase II; CaMV35S(3×) cauliflower mosaic virus 35S promoter; OSC, octopine synthase; SP, Avr4 signal peptide; HIS, RGS histidine tag; DrsB1, dermaseptin B1; CBD, carbohydrate binding domain from Avr*4* effector protein; L1, helix-forming linker (EAAAK)4; L2, linker sequence from *Caenorhabditis elegans* chitinase (CCD73759) gene; RB and LB, right and left borders of Agrobacterium T-DNA region. Two CBDs are fused together by a linker sequence from *Caenorhabditis elegans* chitinase (CCD73759) gene.

The DNA coding sequence of DrsB1 mature peptide (GenBank accession P80282) encoding 31 amino acid (AMWKDVLKKIGTVALHAGKAALGAVADTISQ) was fused to *C. fulvum* Avr4 effector protein chitin-binding domain, CBD (GenBank accession CAA69643.1). CBD was fused to DrsB1 peptide in both N- and C-terminus by the rice chitinase helix-forming linker (EAAAK)4 (GenBank accession No.: X54367.1). *Nco*I and *Bam*HI restriction sites were designed in the 5′ and 3′ termini of the construct for cloning purposes, respectively. Furthermore, two repeats of CBDs fused together by a linker sequence from *Caenorhabditis elegans* chitinase (CCD73759) gene were also fused to the N-terminal of DrsB1. An RGS-His tag was engineered to the N-terminal of the recombinant genes for purification purposes. The entire fusion peptides were commercially synthesized in the pUC57 cloning vector (Biomatik, Canada). The codon optimization was based on the codon-usage bias of the host tobacco plant (http://www.jcat.de). The recombinant genes were then digested with *Nco*I and *Bam*HI restriction enzymes, and ligated into pre-digested pGSA1285 binary expression vector, resulted in three expression vectors, namely pGSA/CBD-DrsB1, pGSA/DrsB1-CBD, and pGSA/(CBD)2-DrsB1 (**Figure 1**). The molecular weight of mature recombinant peptide excluding the signal peptide (SP) was 13.2, 13.2, and 23.1 kDa, respectively. Next, binary vectors were transformed into competent *A. tumefaciens* (GV3101) cells by electroporation (2,400 V, 200 Ω, and 25 μF). Details of mature expression cassettes are shown in **Supplementary Figure 1**.

### Plant Materials

The seeds of tobacco (*Nicotiana tabacum *L. var Xanthi) plant were obtained from the Thirtash Research and Education Center (Mazandaran, Iran). Seeds were initially surface sterilized by soaking in 70% (v/v) ethanol for 30 s and then washed three times with distilled water to remove ethanol residues. Seeds were subsequently soaked in 1,400 μl detergent solution (including 450 μl 2% sodium hypochlorite, 900 μl distilled water, and 50 μl Triton X-100) for 15 min. Finally, seeds were rinsed five to seven times, using sterile distilled water and were grown on MS medium (Murashige and Skoog, 1962) supplemented with 30 g/L sucrose, 7 g/L agar for 6–8 weeks at 25 ± 2°C with a 16/8-h light/dark photoperiod.

### Generation of Transgenic Tobacco Plants

Leaf discs (1 cm^2^) of the tobacco plants were transformed by *A. tumefaciens* harboring binary vectors for 10 min with gentle shaking. The explants were dried on sterile filter paper, then inoculated leaf discs were transferred to the solid basal co-cultivation medium containing 0.1 mg/L naphthaleneacetic acid (NAA) and 2 mg/L 6-Benzylaminopurine (BAP) and 30 g/L sucrose. Leaf discs were then placed on a selection medium containing 0.1 mg/L NAA, 2 mg/L BAP, 100 mg/L cefotaxime, and 50 mg/L Kanamycin for regeneration and shoot formation. Regenerated adventitious shoots were transferred to a selection MS medium supplemented with 0.1 mg/L NAA, 2 mg/L BAP, and 100 mg/L Kanamycin. Shoots that survived were then placed on a rooting medium containing 2 mg/L IBA, 0.5 mg/L NAA, and 100 mg/L kanamycin. After 4 weeks, plants were transferred to sterile soil (50% soil and 50% coco peat and perlite) for growth in the greenhouse at 25°C, 8/16-h dark/light cycle, and 50% humidity conditions for disease analysis.

### DNA Extraction and Screening of Putative Transgenic Lines

To ensure integration of transfer DNAs (T-DNAs) in putative transgenic plants, genomic DNA was extracted from putative transgenic lines and a non-transgenic plant (Ut), using cetrimonium bromide (CTAB) method (Richards et al., 1994). Next, integration of the transgenes was confirmed by PCR analysis, using transgenes specific primers.

### Semi-Quantitative Real Time-PCR Analysis of Transgenic Lines

Total RNA was extracted from 200 mg of young leaves of selected PCR positive transgenic lines, and a non-transgenic plant, using lithium chloride method (Chang et al., 1993). The quality and quantity of extracted RNAs were determined using 1% agarose gel electrophoresis and spectrophotometry, respectively. To remove DNA, total RNA was treated by DNase I (Thermo Scientific) and complementary DNAs (cDNAs) were synthesized using a first-strand cDNA synthesis kit (Thermo Scientific) according to the manufacturer’s instructions. Semi-quantitative real time-PCR (RT-PCR) analysis was performed using DrsB1 specific primers (DrsB1_F: 5´-GCTAAGGCTATGTGGAAGGATG‐3′´; DrsB1_R: 5´-ATTGAGAAATAGTATCAGCAACAGC-3´). The elongation factor 1α (elf1α) primers (elf1α_F:5´-TCTTAACCATACCAGCATCACC-3´; elf1α_R:5´-TGAACCATCCAGGACAGATTG-3´) were used as an internal control. The semi-quantitative RT-PCR reaction was performed as follows: pre-denaturation at 94°C (5 min), then 40 cycles at 94°C (60 s); 59 or 58°C (30 s) and 72°C (30 s), and a final extension at 72°C for 10 min. The RT-PCR products were electrophoresed and compared on 1.5% agarose gel.

### Protein Extraction

Total protein was extracted from the selected transgenic and non-transgenic plants using phosphate buffer (50 mM, pH 7) containing 1 mM phenylmethylsulfonyl fluoride (PMSF) protease inhibitor (Roche, cOmplete, Mini, Germany). Briefly, 500 mg leaf tissue (FW: fresh weight) was ground in liquid nitrogen, and the powder was suspended in 1:1 (w/v) phosphate buffer and vortexed vigorously for 2–5 min. Extracts were centrifuged at 13,000 rpm for 30 min at 4°C and supernatants were filtered using a 0.45-μm pore size filter. The concentration of total crude protein extracted from selected transgenic and control plants was determined using the Bradford method (Bradford, 1976) with bovine serum albumin (BSA) as the standard protein.

### Purification of the Recombinant Peptides and Western Blot Analysis

The recombinant peptides were purified using a chromatography column containing the PrepEase Ni-IDA resin (USB Corporation) from selected transgenic lines. Briefly, the column was equilibrated with loading 600 µl of 1× LEW buffer (50 mM NaH_2_PO_4_, 300 mM NaCl, pH 8) and then, up to 600 µl of total proteins from transgenic plants were loaded onto the column. Next, the columns were washed twice with wash buffer (50 mM NaH_2_O_4_, 300 mM NaCl, pH 8). Finally, the purified recombinant peptides were removed from the column by the rinsing solution containing imidazole (50 mM NaH_2_PO_4_, 300 mM NaCl, 250 mM imidazole, pH 8). Purified recombinant peptides concentration was determined using the Bradford method (Bradford, 1976) with bovine serum albumin (BSA) as the standard protein.

Purified recombinant peptides were then electrophoretically separated on a 14% polyacrylamide gel at 150 V and blotted on a nitrocellulose membrane (Mini-PROTEAN II Multiscreen Apparatus, Bio-Rad). The nitrocellulose membrane was blocked for 1 h with tris buffered saline (TBS) containing 5% powdered milk. The nitrocellulose membrane was washed three times with the TBS buffer and then exposed to 1:2,000 dilution of mouse monoclonal anti-His antibody at 37˚C for 1 h. The nitrocellulose membrane was then washed three times with the TBS buffer and then exposed to 1:2,000 dilution of mouse monoclonal anti-His antibody at 37°C for 1 h followed by 3,3´-diaminobenzidine tetrahydrochloride (DAB) detection according to the manufacturer’s instructions.

### Phytopathogens, Culture, and Maintenance

The following phytopathogenic microorganisms were used to evaluate the antimicrobial activity of the recombinant peptides: fungi were cultured on potato dextrose agar (PDA) plates (200 ml boiled potatoes; 15 g/L dextrose; 20 g/L agar, and 800 ml deionized water) in low light at ambient temperature. For antifungal assays, the spores were collected and suspended in potato dextrose broth (PDB) Sigma–Aldrich, Inc., St. Louis, MO). Spore concentrations were determined by using a hemocytometer under a light microscope.

### Antifungal Assays

#### Antifungal Activity of Total Protein From Transgenic Plants

The antifungal activity of total protein extracted from selected transgenic and non-transgenic lines was determined against *F. oxysporum, F. solani, A. solani*, *A. alternata, P. aphanidermatum*, and *Pythium* sp. fungi according to Khademi et al. (2019). Total protein extracted from transgenic and non-transgenic plants was mixed with sterile PDA to the final concentration of 50 μg/ml. The PDA medium was then poured into 90 mm petri plates. Next, 10 mm plug from fresh fungal cultures was placed in the center of the plates and cultures were incubated for several days as needed for different fungi at 25°C. After 5 days, the diameter of the fungal growth was measured in three directions and averaged. Mycelium growth inhibition was calculated as the percentage of inhibition of radial growth relative to the control (Feng and Zheng, 2007).

To evaluate fungal minimum inhibitory concentration (MIC) values as the lowest concentration of recombinant peptides, that prevents visible growth of microbes, various concentrations (7.5, 10, 12.5, 15, 17.5, 20, 22.5, 25, 27.5, and 30 µg/ml) of the purified recombinant peptides were mixed with the fungal spore suspension (1×10^8^ CFU/ml) in a final volume of 100 µl. After 24 h, germinated conidia and spores were counted under a light microscope. The MIC values were defined as the lowest concentration of recombinant peptides required for complete suppression of fungal spore and conidia germination (Yevtushenko et al., 2005). Moreover, the IC50 was also measured from the corresponding dose–response curves for each peptide-pathogen combination (Yevtushenko and Misra, 2007). To ensure the assay measurements, a completely randomized design with three replications was used.

### Detached Leaves Resistance Bioassays

To evaluate resistance of detached leaves to *F. oxysporum, F. solani, A. solani*, *A. alternata, Pythium aphanidermatum*, and *Pythium* sp. fungi, experiments were performed as described previously by Osusky et al. (2005) and Yevtushenko and Misra (2007). Five to seven centimeter in length tobacco leaves from selected transgenic lines and non-transgenic tobacco plants each in triplicates were excised from 5-week-old plants and were placed in petri dishes on wet filter papers. Next, a 1 cm ^2^ agar block of freshly grown fungi cultures was directly placed on detached leaves. The leaves were co-cultivated with fungi under standard plant growth conditions and observed daily for disease development including blight, brown leaf spot, leaf dehydration and chlorosis, water-soaking, and decayed tissue for 4 weeks (Osusky et al., 2005; Yevtushenko and Misra, 2007). Disease severity (%) was measured as the sum of leaves area showing symptoms to total leaves area. Data were analyzed by ANOVA and means were compared by either by least significant differences (LSD) or Duncan’s multiple range test (P ≤ 0.01) by using SAS 9.1 (SAS, Inc., North Carolina, USA) software.

### Transgenic Whole Plants Resistance Bioassays

To evaluation the resistance behavior of whole transgenic plants, 5-week-old selected transgenic lines and control tobacco plants were challenged with the pathogens as described previously by Yevtushenko et al. (2005) and Osusky et al. (2005). Briefly, CBD-DrsB1-38, (CBD)2-DrsB1-43, and DrsB1-CBD-08 transgenic lines and a control tobacco plant were challenged with *F. oxysporum, F. solani, A. solani*, *A. alternata, P. aphanidermatum*, and *Pythium* sp. fungi. Two agar blocks (1 cm^2^) fully covered with fungal mycelia were placed 2 cm from the stem of a well- developed tobacco plant grown *in vitro*. During the co-cultivation period, development of disease symptoms was recorded daily and plant resistance was assessed based on the emergence of symptoms intensity, including yellowing and wilting of leaf veins, curly top and/or dark spots, brown stems, and plant collapse.

### Disease Resistance Analysis of Transgenic Plants Under Greenhouse Conditions

To test the resistance of transgenic tobacco plants to *F. solani, F. oxysporum*, and *Pythium* sp., selected transgenic lines were challenged with fungi inoculum. Briefly, for *F. oxysporum* and *F. solani* disease resistance test, roots of 2-weeks-old selected transgenic and non-transgenic lines were dipped into a spore suspension (adjusted at 10^6^ spores per milliliter) for 1 min. Plants were then planted into pots for 4 weeks in a growth chamber in a 16-h photoperiod at 23 ± 2°C at 70–80% relative humidity.

For *Pythium* sp. bioassay, 3-week-old transgenic and non-transgenic control plants were inoculated with a 3-mm-diameter disc of mycelium isolated from 7 days-old *Pythium* sp. culture. Plants were grown under optimum humidity and temperature conditions in the same growth as described above. The experiment was repeated three times and resistance symptoms developments was recorded. Shoot height, root height, stem fresh weight, root fresh weight, shoot dry weight, and root dry weight of transgenic and non-transgenic plants were recorded and analyzed with one-way ANOVA. Unfortunately, greenhouse conditions was not appropriate for inoculation and disease development by *Alternaria* species at our laboratory.

### Scanning Electron Microscopy Analysis

Scanning Electron Microscopy (SEM) micrographs were generated to observe and confirm the antifungal effects of the recombinant peptides on the structure of fungal mycelia. Plugs (2 cm^2^) of fresh *F. oxysporum, F. solani, A. solani*, *A. alternata*, *P. aphanidermatum*, and *Pythium* sp. fungi on PDA cultures were prepared to fix the samples and separately treated with extracted proteins (50 μg/ml) from the selected transgenic lines [DrsB-CBD-08, CBD-DrsB1-38, and (CBD)2-DrsB1-43] and extracted protein from non-transgenic tobacco plants and phosphate buffer (as the negative control) for 24 h at 25˚C. Samples were then frozen at −80˚C and dried using freeze-drying machine. After sample fixation, mycelial samples were prepared to be coated with gold particles using a sputter coater under a vacuum evaporator. The coated samples were analyzed using SEM (MIRA3-LMU, USA at HV=20 kV).

### Hemolytic Assay

The hemolytic activity of recombinant peptides was determined against human fresh blood (donated by the Nour Medical Laboratory, Khooramabad). Briefly, human fresh blood was taken directly onto a syringe containing 50 mM ethylenediaminetetraacetic acid (EDTA) and washed three times with phosphate-buffered saline (PBS: 50 mM sodium phosphate, 150 M NaCl, pH 7.0). Next, 100 µl of red blood cells (10^8^ cells per ml) in PBS were plated into 96-well plates, and incubated at 37˚C for one and 24 h mixed with 100 µl of 0.4% Tween-20 (positive control), or 100 µl of PBS (negative control), or 100 µl of diluted purified recombinant peptides (final concentrations of 11.25 to 720 µg/ml) in each well. Next, the released hemoglobin was monitored at 540 nm (Mor and Nicolas, 1994).

### Statistical Analysis

Prior to analysis of variance (ANOVA), the error normality was verified by the Shapiro-Wilk tests by SAS 9.1 (SAS, Inc., North Carolina, USA) software at 5% probability. When significant interactions were observed, LSD was used to compare the mean values. All analysis were evaluated at the 0.05 or 0.01 significance level as indicated.

## Results

For each construct, 350 leaf discs were used for *Agrobacterium*-mediated transformation of which 150 seedlings were regenerated and transferred to the rooting medium. For each transformation event, 20 putative transgenic lines rooted well on the selection medium containing 100 mg/L Kanamycin were selected for further analysis. PCR analysis resulted in amplification of the expected fragments with the right size providing evidences that the T-DNAs encoding the recombinant peptides had been incorporated into the transgenic lines genome. PCR positive transgenic tobacco lines were named CBD-DrsB1-xx, DrsB1-CBD-xx, and (CBD)2-DrsB1-xx based on the type of the construct employed to express the recombinant peptides, xx represents the transgenic line number.

### Semi-Quantitative Real Time-PCR Analysis

**Figure 2:**
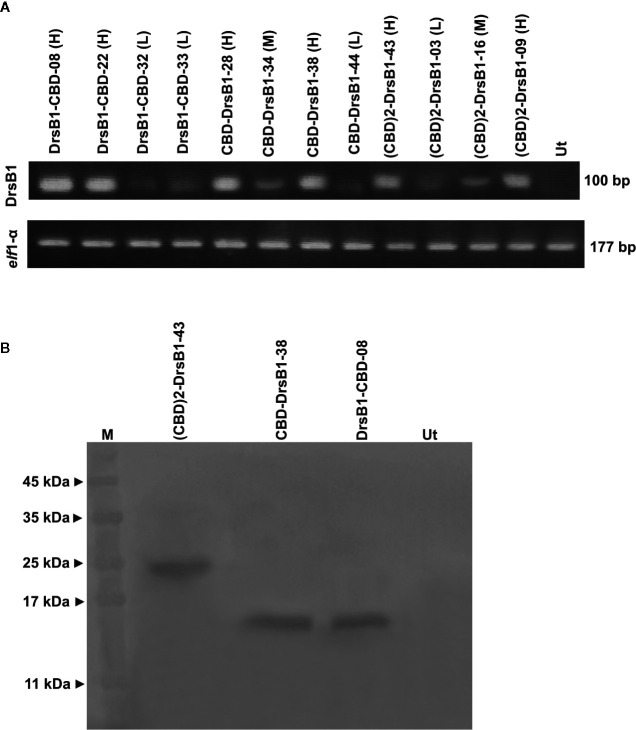
Semi-quantitative RT-PCR **(A)** and western blotting **(B)** analysis of transgenic and control plants. The upper panel shows the PCR products of the CBD-DrsB1, (CBD)2-DrsB1, and DrsB1-CBD recombinant genes in the selected transgenic line and the lower panel shows the PCR products *elf* housekeeping gene. Mouse monoclonal anti-His antibody (Sigma-Aldrich, product No. A7058) was used for recombinant peptides detection.

All selected transgenic plants were thoroughly screened to ensure reliable transgene expression. To this end, and in order to test and compare the transcription level of transgenes in transgenic tobacco plants, the semi-quantitative RT-PCR analysis was performed. As shown in **Figure 2A**, transgenes are transcribed and expected fragments were amplified in the transgenic tobacco lines but not in the non-transgenic tobacco plants, suggesting that the recombinant peptides were successfully transcribed. Selected transgenic tobacco lines were classified as low (L), medium (M), and high (H) expression based on the transcription level of transgenes relative to that of the *elf*1α as the housekeeping reference gene (**Figure 2A**).

To provide evidence for expression of recombinant transgene at the protein level, three transgenic lines with the highest messenger RNA (mRNA) transcript level representing each construct were analyzed by using Western blotting analysis. Protein bands of almost 23, 13, and 13 kDa were found corresponding to (CBD)2-DrsB1, CBD-DrsB1, and DrsB1-CBD recombinant peptides, respectively, which corresponded well with the predicted molecular weight (excluding the signal peptide) of recombinant peptides (**Figure 2B**). These findings suggested that the recombinant peptides have been correctly expressed in transgenic lines. No traces of such proteins were found in the non-transgenic control line (**Figure 2B**).

### The Recombinant Peptides Show Antifungal Activity

**Figure 3:**
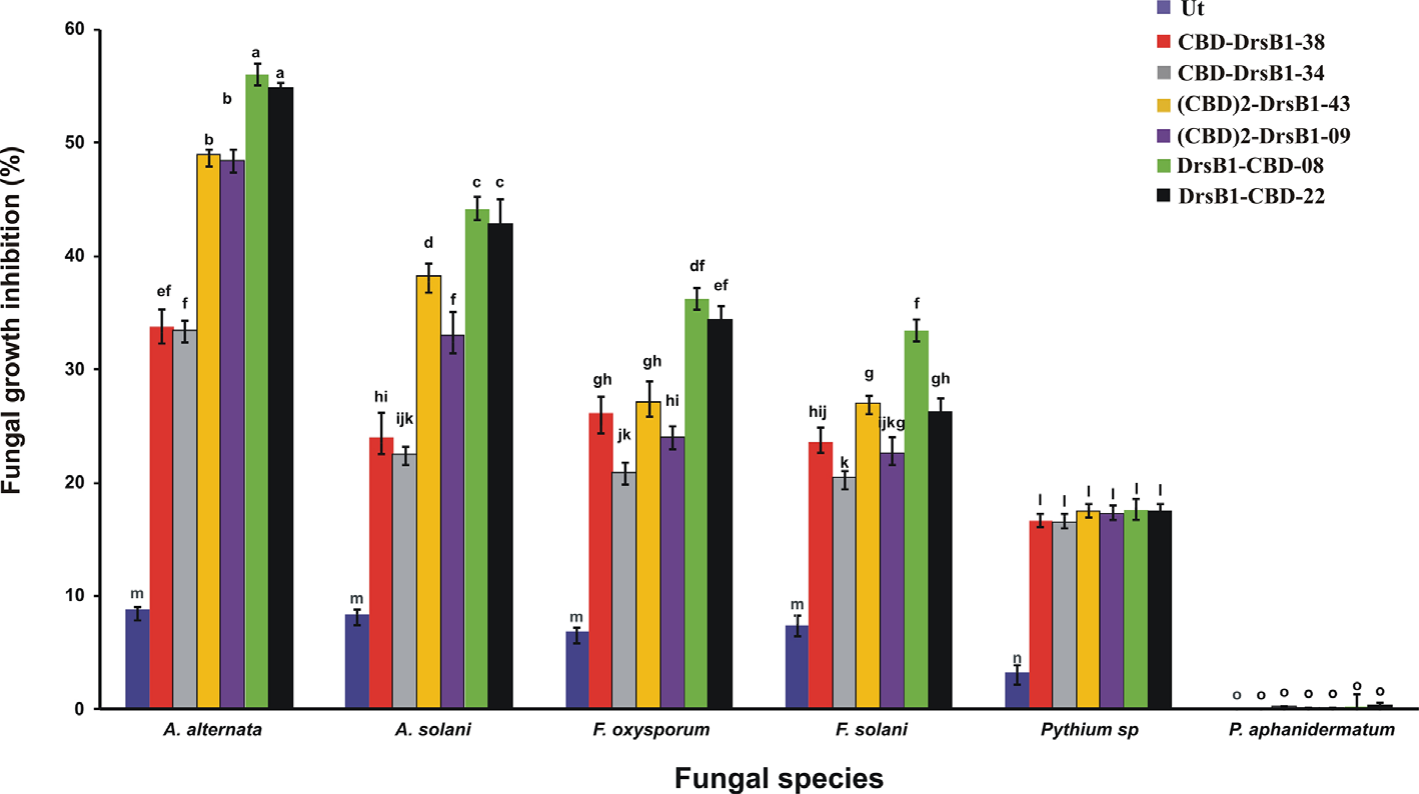
The diagram showing the effect of recombinant peptides on fungal growth. P value of 0.01 was considered as significant; 50 µg/ml of transgenic and non-transgenic total protein was used. Percentage of inhibition was calculated relative to the phosphate buffer treatment. The antifungal activity data were statistically analyzed and the mean comparison was done by least significant differences (LSD) test (P ≤ 0.01) test in three replicates using SAS 9.1 (SAS, Inc., North Carolina, USA) software.

Total protein from selected transgenic ranged from 45 ± 2.1 to 50 ± 1.7 mg/kgFW. Regardless of type of recombinant peptides, the concentration of the recombinant peptides also ranged from 5.5 ± 0.7 to 6.0 ± 0.4 µg/gFW. In order to evaluate the antifungal activity of recombinant peptides *in vitro*, protein extracts from selected transgenic and control plants were used against a number of pathogenic fungi. Analysis of variance of inhibition of fungal growth demonstrated that recombinant peptides had a significant (P<0.01) inhibition effect on fungal mycelium growth (**Supplementary Table S1**). **Figure 3** compares the growth inhibition means of different fungi challenged with protein extracts. All three recombinant peptides had the highest and the lowest inhibitory effect against *A. alternata* and *P. aphanidermatum*, respectively (**Figure 3**). As it can be seen from **Figure 3**, DrsB1-CBD recombinant peptide had the highest activity against all fungi tested, except *Pythium* species, suggesting the orientation of the fusion proteins matters.

**Figure 4:**
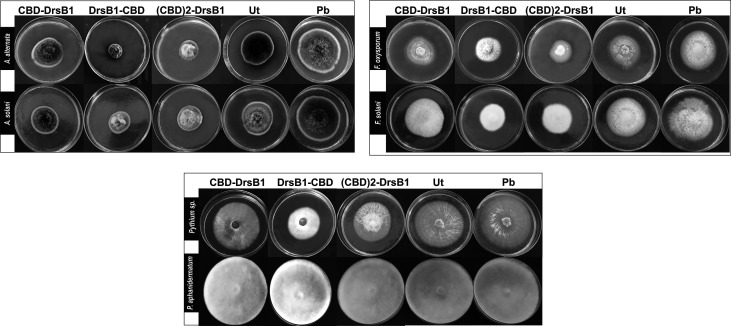
Hypha extension inhibition assay by mixing recombinant peptides with the culture medium. Fungi were cultured on potato dextrose agar (PDA); 50 μg/ml of total protein from the non-transgenic line (Ut) and sterilize phosphate buffer (Pb) were used as the negative controls.

To find the lowest concentration that completely inhibited the growth of pathogens, the MIC of recombinant peptides extracted from selected transgenic lines was measured (**Table 1**). The MIC for fungi varied greatly ranged from to >30 µg/ml. The recombinant peptides had a greater inhibitory activity against *A. alternata* and *A. solani*, followed by *Fusarium* species. Recombinant peptides had no inhibitory effect on *P. aphanidermatum* even at 30 µg/ml. Interestingly, the results of mixing the recombinant peptides with the fungal culture media were in good agreement with that of MIC and IC50 (**Table 1** and **Figure 4**). It is tempting to say that the MIC for DrsB1-CBD and (CBD)2-DrsB1 peptides was lower than that of CBD-DrsB1 (**Table 1**).

**Table 1:** Antifungal assay of recombinant peptides against fungal plant pathogens.

Pathogen	CBD-DrsB1-38		CBD-DrsB1-28		(CBD)2-DrsB1-43		(CBD)2-DrsB1-09		DrsB1-CBD-08		DrsB1-CBD-22		DrsB1-04	
	MIC	IC50	MIC	IC50	MIC	IC50	MIC	IC50	MIC	IC50	MIC	IC50	MIC	IC50
*A.* ***alternata***	20	8.75	20	9.01	12.5	5.74	12.5	6.61	10	5.38	10	5.73	22.5	11.71
*A. solani*	20	9.73	20	9.96	12.5	6.79	12.5	6.88	10	6.35	10	6.56	22.5	12.18
*F. solani*	25	13.52	25	13.63	17.5	9.38	17.5	9.64	15	9.22	15	9.26	25	14.58
*F. oxysporum*	25	10.16	25	10.21	17.5	8.37	17.5	8.97	15	8.14	15	8.98	25	13.15
*Pythium* sp.	30	14.45	30	14.77	22.5	10.76	22.5	10.70	20	9.98	20	10.15	30	14.45
*P. aphanidermatum*	>30	–	>30	–	>30	–	>30	–	>30	–	>30		>30	–

MIC, minimum inhibitory concentration (µg/ml); IC50, the half maximal inhibitory concentration (µg/ml).

### Hemolytic Activity Assay

To address the phytotoxicity of recombinant peptides, the viability of erythrocyte cells was evaluated in the presence of recombinant peptides (**Supplementary Figure S2**). The hemolysis activity of recombinant peptides at various concentrations (11.25–720 µg/ml) of the purified recombinant peptides showed that the recombinant peptides had no significant hemolytic activity on mammalian erythrocytes even at the highest concentration tested (720 µg/ml).

### Scanning Electron Microscopy Analysis

**Figure 5:**
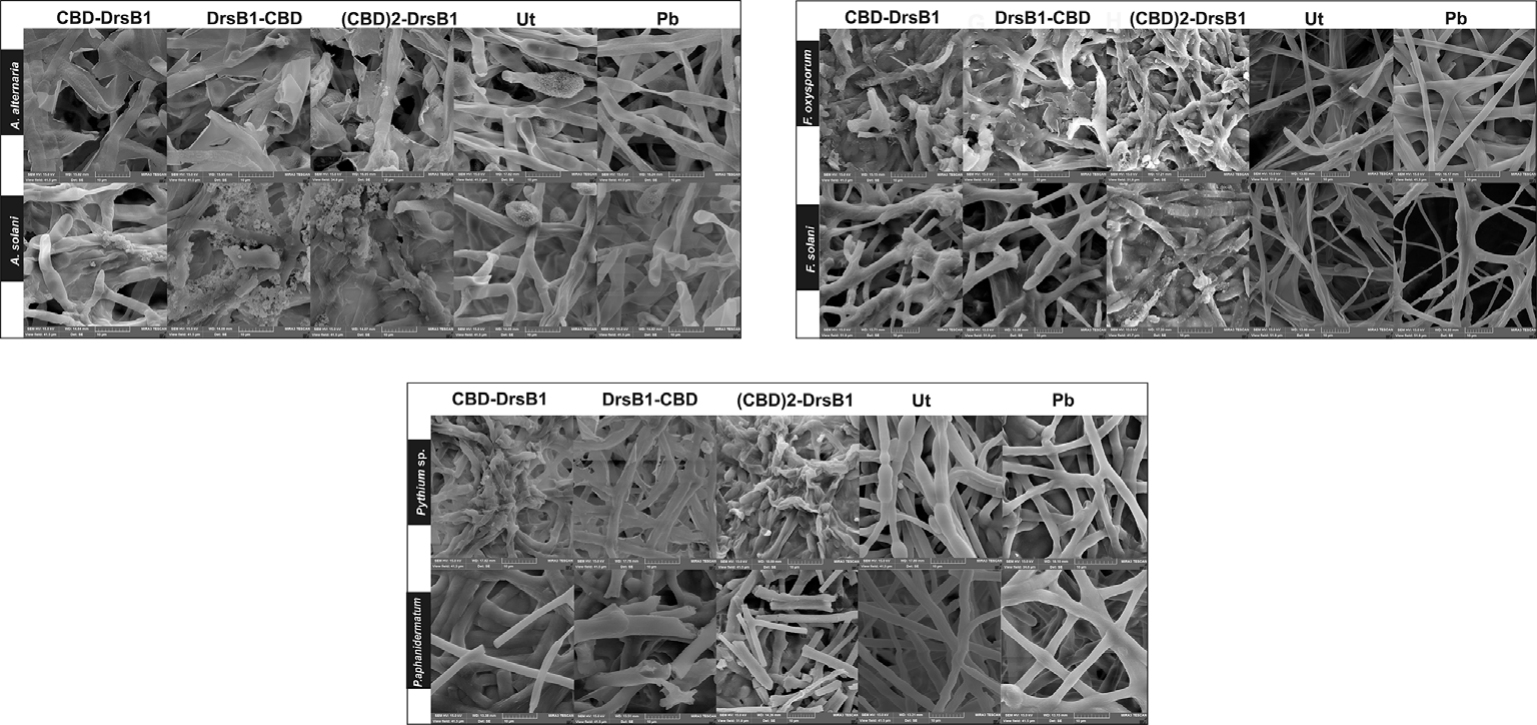
Scanning electron microscopy images of fungal hyphae treated with the total protein (50 μg/ml). Scale bars are indicated in micrometer for each image. Ut, non-transgenic control line; Pb, treated with phosphate buffer as control.

In order to investigate the effect of recombinant peptides activity on fungal pathogens cell wall structures, fungal mycelium and spores were treated with total protein extracted from transgenic lines and a non-transgenic control plant. SEM images revealed that *Alternaria* spores were deformed and apparently burst leading the cell contents to spill (**Figure 5**). Furthermore, numerous adhesions and ruptures were observed in the structure of the fungal mycelium. It seems that DrsB1-CBD and (CBD)2-DrsB1 recombinant peptides had a greater destructive effect on fungal hyphae and spores compared with that of the CBD-DrsB1 recombinant peptide. As it can be seen from **Figure 5**, fungal mycelia treated with phosphate buffer and non-transgenic plants total protein extracts, had normal, smooth, and intact structures, whereas shrunken and wrinkled spores and hyphae were seen following treatment with the protein extracts from transgenic plants. Generally speaking, all three recombinant peptides had similar effects on the structure of *Pythium* sp. mycelia (**Figure 5**). The effect of the recombinant peptides on *Fusarium* cells was also noticed as folding, twisting, adherence compared to the effect of phosphate buffer treatment and total proteins from non-transgenic control plants (**Figure 5**). These observations suggest that the antimicrobial effects of the recombinant peptides are directly related to the structural damage to both fungal spores and mycelia.

### Enhanced Disease Resistance of Transgenic Tobacco Plants Under *In Vitro* Conditions

To evaluate if expression of recombinant peptides can confer resistance to fungal infections, detached leaves and *in vitro* plants were challenged with few devastating fungi. Necrotic lesions on control leaves were noticed the same day after inoculation. Detached leaves from CBD-DrsB1, (CBD)2-DrsB1, and DrsB1-CBD transgenic plants showed chlorosis symptoms with 2 days and 3 days delay in comparison to non-transgenic plants, respectively.

**Figure 6:**
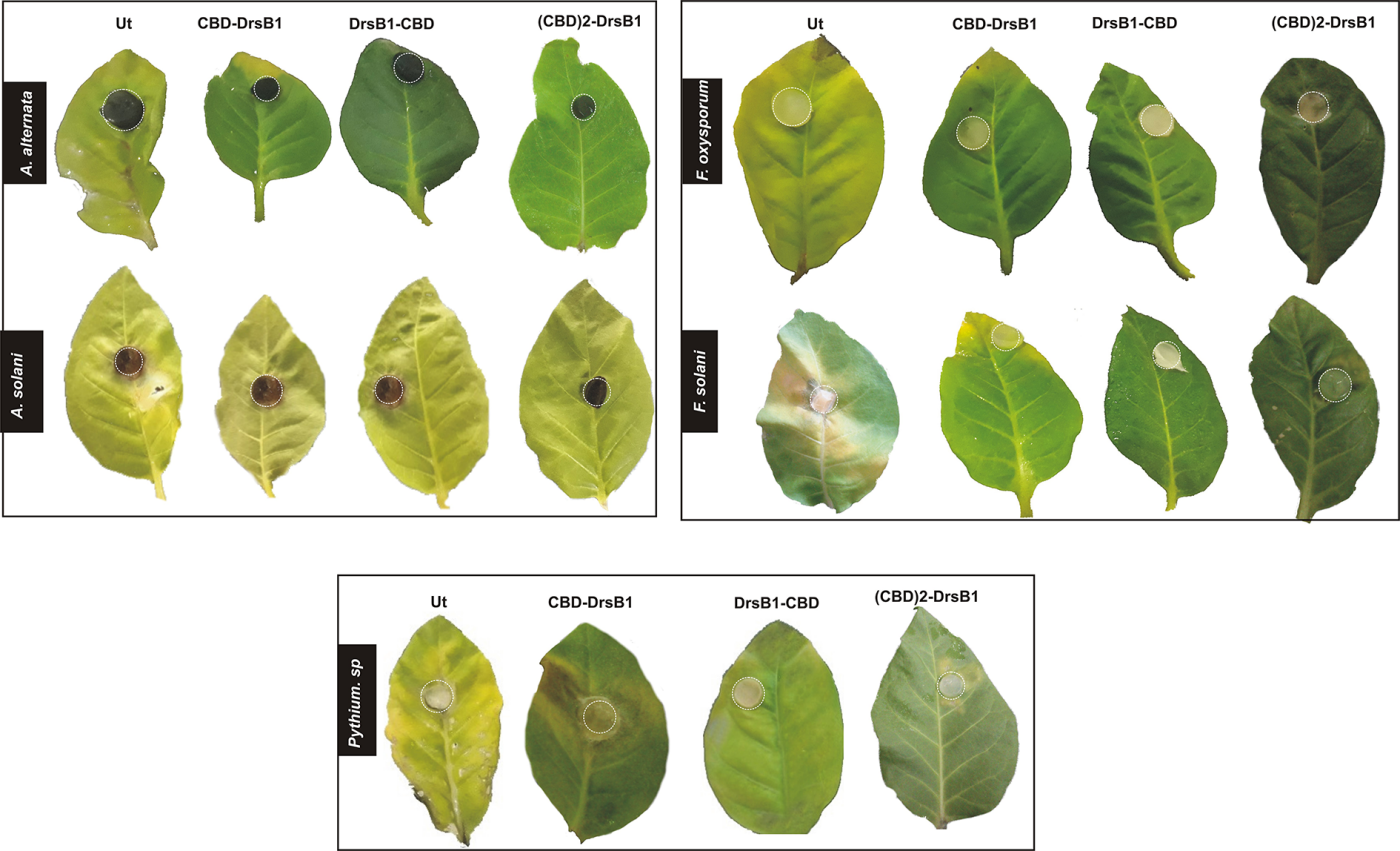
Detached leaves assay for the fungal disease incidence in different transgenic lines and control tobacco plants challenge with different fungi. Dotted circles indicate the inoculation points with a 1-cm^2^ agar block of the fungal culture, control and transgenic lines, 21 days post-incoulation with *Alternaria*, control and transgenic lines, control and transgenic lines, 11 days post-inoculation with *Fusarium*. 7 days post-inoculation with *Pythium* sp.

The severity of the infection by *P. aphanidermatum* was high as compare to other fungi in such that all leaves, including transgenic leaves displayed severe necrosis, withered, and rotted 5 days after inoculation (**Figure 6**). Noticeable disease symptoms including chlorotic and necrotic spots were found on detached leaves 3 days after inoculation with *Pythium* sp. (**Figure 6**). Seven days after inoculation, the transgenic leaves became necrotic, whereas un-transformed control leaves showed extensive brownish spots 3 days after inoculation.

**Figure 7:**
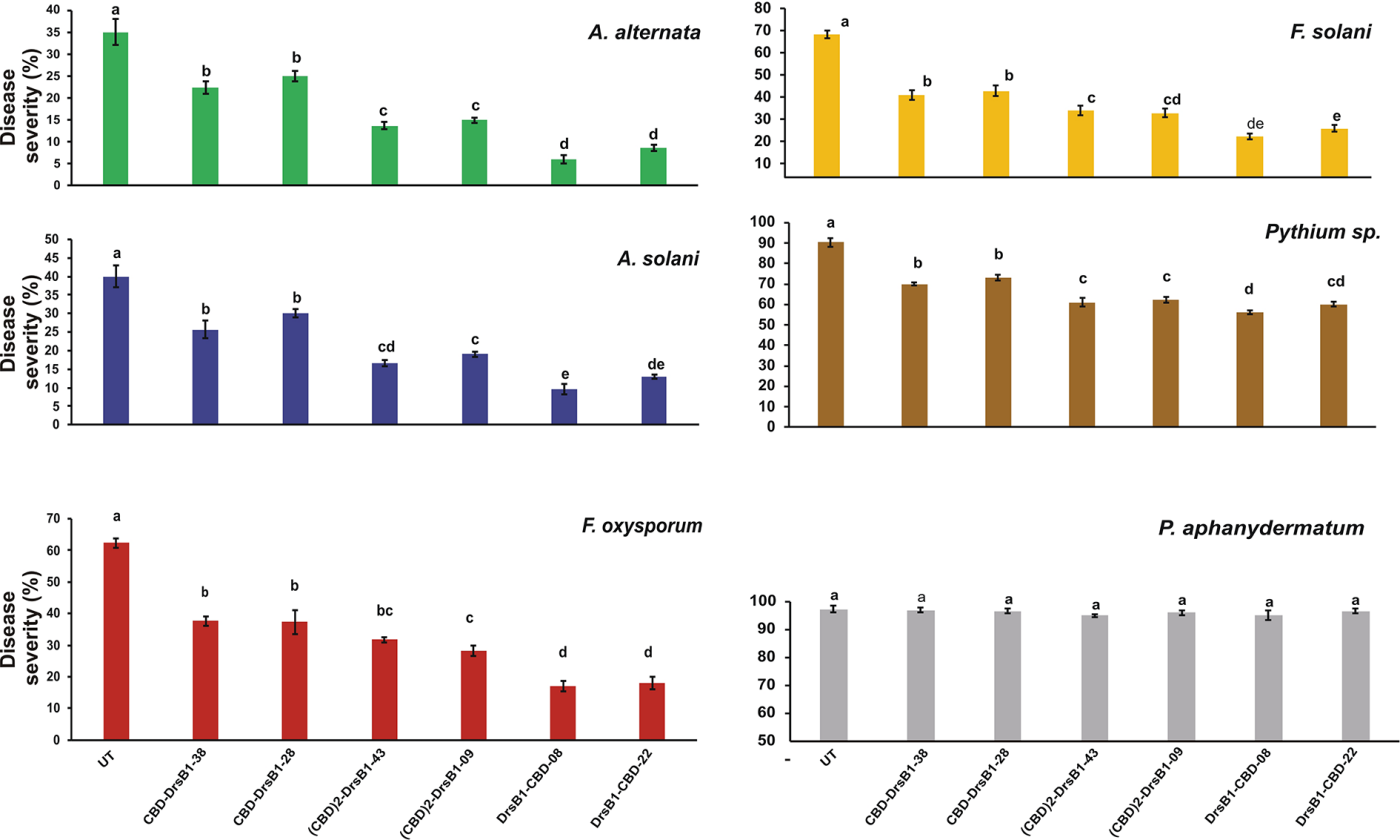
The diagram showing the disease severity of the detached leaves. The disease severity is calculated based on the ratio of the damaged area to the total leaf area. The antifungal activity data were statistically analyzed as a completely randomized design in a 6×7 factorial arrangement and the mean comparison was done by least significant differences (LSD) (p ≤ 0.01) test in three replicates using SAS 9.1 (SAS, Inc., North Carolina, USA) software. The p value of ≤0.01 was considered as significant.

Hypersensitivity reaction (HR) symptoms resulted in necrosis spots following *F. solani* and *F. oxysporum* infections. As compared to other fungi tested in this study, transgenic plants were more resistant to *A. alternata* and *A. solani.* Interestingly, 3 weeks after inoculation, only small necrotic lesions were observed at the site of inoculation with *A. alternata* and *A. solani*. The lowest severity of chlorosis or necrosis symptoms was observed in transgenic lines expressing DrsB1-CBD and (CBD) 2-DrsB1, respectively (**Figure 7**). In contrast, leaves from CBD-DrsB1 transgenic plants showed extensive areas of necrosis spots surrounded by chlorotic halos (**Figure 7**). Consistent with observations, quantitative data on disease severity (%) showed that transgenic lines resistance was significantly different with respect to the expressed recombinant protein (**Supplementary Table S2**). As it can be seen, transgenic line had the lowest and the highest resistance against *Pythium* species and *Alternaria* species, respectively (**Figure 7**).

### *In Vitro* Plants Are Disease Resistance to Fungal Infections

**Figure 8:**
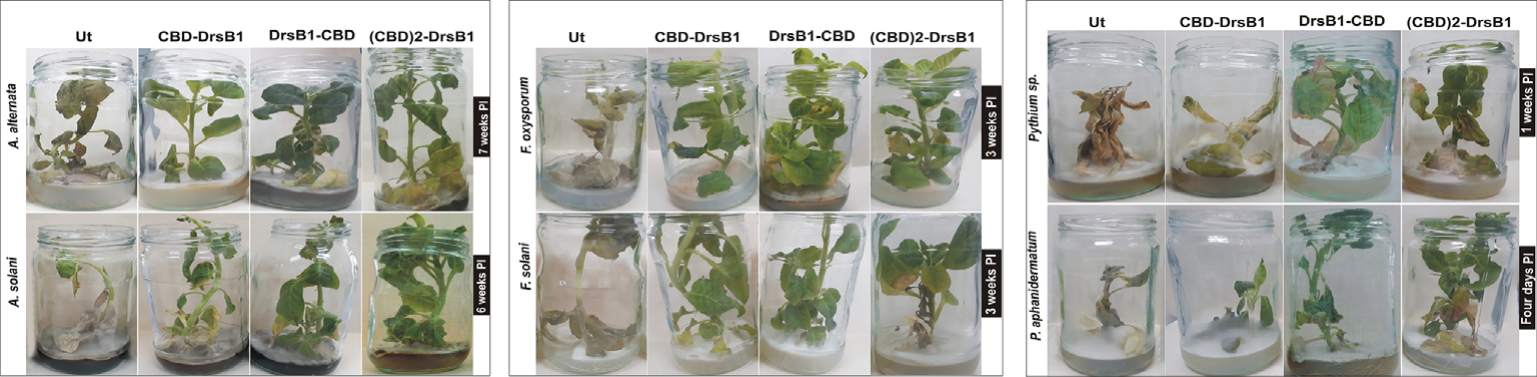
Resistance of transgenic tobacco (*Nicotiana tabacum* cv. Xanthi) plants expressing the recombinant peptides constructs in jars inoculated with different fungi.

To evaluate the disease resistance of *in vitro* grown plants, selected transgenic plants and non-transgenic control plants in jars were challenged with fungal mycelia. Plants were evaluated based on the pathogenicity of the fungi and symptoms developments, including discoloration and chlorosis, necrosis, wilt, and the number of days of survival after inoculation. Depending on fungus species, emergence of visible disease symptoms varied from 3 days to 2 weeks for *P. aphanidermatum* and *A. alternata*, respectively. In contrast to transgenic plants, symptoms of the diseases such as discoloration of leaves and rotting the entire plant occurred within a shorter period of time for non-transgenic plants (**Figure 8**). Seven days after inoculation with *F. solani* and *F. oxysporum*, symptoms of wilting, dieback, chlorosis, and necrosis appeared on non-transgenic plants resulting in severe wilting and death in less than 12 days. Interestingly, symptoms of the diseases appearance varied from 19 to 28 days after inoculation for CBD-DrsB1-38, DrsB1-CBD-08, and (CBD)2-DrsB1-43 lines, respectively. Among the transgenic lines, DrsB1-CBD-08 showed the least disease severity compared to other transgenic lines. (CBD)2-DrsB1-43 transgenic line was moderately resistant and disease symptoms appeared with 5 days delay as compared to CBD-DrsB1 transgenic line. **Figure 8** shows significant resistance of transgenic lines expressing recombinant peptides to *A. alternata* and *A. solani* fungi. After 2 weeks of incubation, symptoms of wilting, burns, and necrosis appeared on the non-transgenic control plants with bending down 3 weeks after inoculation (**Figure 8**), whereas transgenic plants expressing recombinant peptides remained green and did not show any signs of disease during this period.

Three days after inoculation with *P. aphanidermatum*, mycelia covered the surface of MS medium and infiltrated the roots and shoots of control plants. The non-transgenic plants were infected from the roots to the tips 4 days after inoculation resulting in yellowish leaves, eventually control plants died in 4 days (**Figure 8**). In contrast, transgenic plants challenged with *Pythium* sp. survived 7 days longer. CBD-DrsB1-38, DrsB1-CBD-08, and (CBD)2-DrsB1-43 transgenic lines showed lower relative resistance to *Pythium* species. Interestingly, after 50 days of inoculation, transgenic plants challenged with *A. alternata*, remained green and healthy (**Figure 8**).

### Enhanced Disease Resistance of Transgenic Tobacco Plants Under Greenhouse Conditions

**Figure 9:**
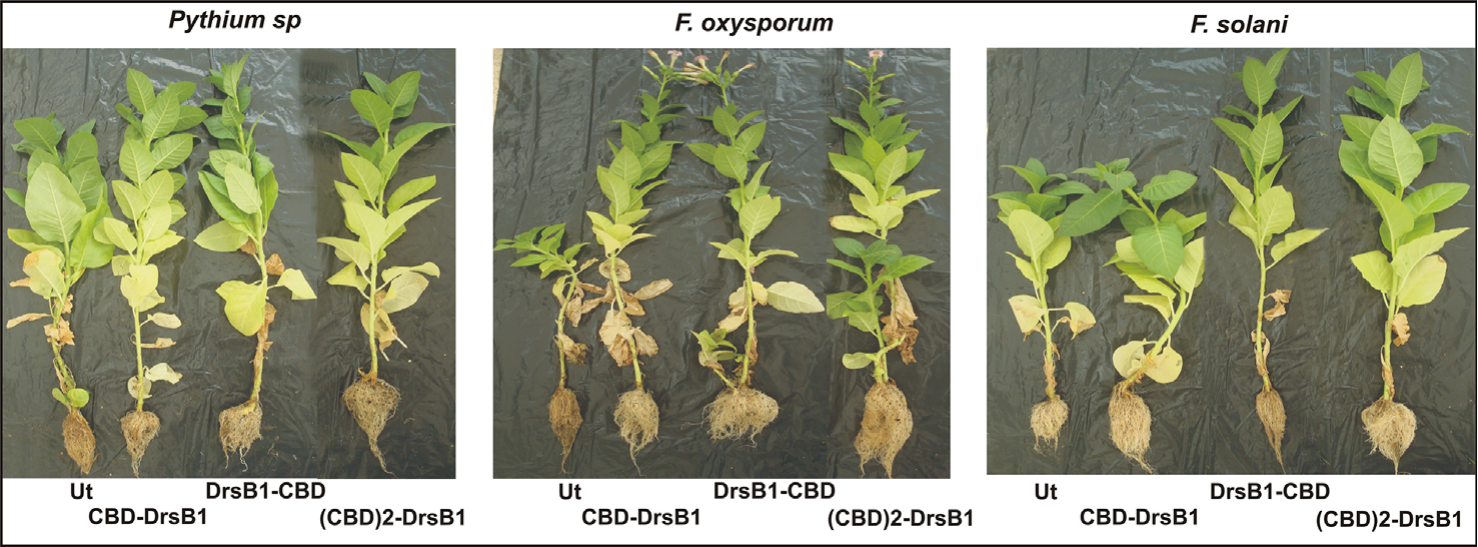
Greenhouse evaluation of transgenic tobacco plants expressing recombinant peptides for resistance against different fungi. Symptoms of fungal disease on transgenic and non-transgenic plants 2 months after inoculation with three fungal species under greenhouse conditions are seen.

In order to investigate the resistance of transgenic plants to fungal infections under greenhouse conditions, selected transgenic lines as well as a non-transgenic control plant were inoculated with *F. oxysporum* and *F. solani*, and *Pythium* sp. fungi. Upon infection, non-transgenic control plants exhibited typical disease symptoms with rapidly leaf discoloration, chlorosis, and leaf death, whereas transgenic plants displayed lesions with reduced size (**Figure 9**). Analysis of variance for main morphological traits (**Supplementary Table S3**) showed that the expression of transgenes resulted in significant (P<0.01) changes in plant morphological characteristics following disease inflicted by fungi. Overall, the transgenic plants showed a better performance with respect to morphological indices (**Figure 9** and **Supplementary Table S3**).

Although it was not possible to challenge transgenic plants with *Alternaria* species and *P. aphanidermatum*, due to unfavorable greenhouse conditions, inoculation of transgenic plants with *Pythium* sp. mycelia resulted in significant decrease of morphological traits compared to non-transgenic control plants (**Figure 9** and **Supplementary Table S3**). DrsB1-CBD-08 and (CBD)2-DrsB1-43 transgenic lines were more tolerant of *Pythium* sp. infection than CBD-DrsB1-38 line, respectively, and a showing a significantly higher shoot length and root biomass (**Figure 9**).

## Discussion

In recent decades, the immense utilization of fungicides to control phytopathogens has contributed to the environmental contamination as well as the emergence of fungicide-resistant phytopathogens (Shattock, 2002). To address these issues, strategies based on the use of molecular genetics to improve disease resistance through the development of transgenic plants are needed (Collinge et al., 2008).

Our earlier works demonstrated that transgenic tobacco plants expressing a recombinant alfalfa peptide gene exhibited increased resistance to fungal and bacterial pathogens (Badrhadad et al., 2018). More recently, Khademi et al. (2019) demonstrated that a dermaseptin B1 fused to either terminus of Avr4 chitin-binding domain, exhibited significant antimicrobial activities when expressed in tobacco hairy roots. Furthermore, a tandem repeat of the same CBD fused to DrsB1 resulted in enhanced antimicrobial activity of the (CBD)2-DrsB1 compared with that of DrsB1 peptide *in vitro* (Shams et al., 2019). Therefore, to test the ability of transgenes in providing protection against plant pathogens, transgenic tobacco plants were generated and challenged with devastating plant pathogens.

The inhibitory activity of natural and synthetic AMPs against pathogenic bacteria and fungi has been demonstrated (Dean et al., 2011). Heterologous expression of AMPs has been successfully reported in many crop plants (Montesinos and Bardaji, 2008; Holaskova et al., 2015). Among diverse group of AMPs, dermaseptins are the most potent member of AMPs and among dermaseptins, dermaseptin B1, is about 20-fold more active than dermaseptin S against filamentous fungi (Osusky et al., 2005). Expression of Dermaseptin B1 in plants has led to enhance plant resistance against a wide range of pathogenic yeast, fungi, oomycetes, gram-negative and gram-positive bacteria (Mor et al., 1994; Mor and Nicolas, 1994; Osusky et al., 2005; Yevtushenko and Misra, 2007; Nicolas and El Amri, 2009; Yevtushenko and Misra, 2012; Yevtushenko and Misra, 2019). To increase the DrsB1 peptide affinity toward microorganisms membrane, Osusky et al. (2005) altered the N-terminal part of DrsB1 peptide and expressed the modified peptide (MsrA2) in potato. Transgenic lines were resistant to *Alternaria*, *Phytophthora, Fusarium, Pythium* fungi, and *Erwinia carotovora* bacterium (Osusky et al., 2005), suggesting that increasing the affinity of DrsB1 toward pathogens membrane, can lead a higher resistance to pathogens. In line with this finding and with regard to the lack of chitin in the plant cell walls, we designed recombinant peptides to develop disease-resistant plants by targeting fungal cell walls. Interestingly, expression of alfAFP peptide fused to rice chitinase CBD in tobacco plants led to plants resistance to pathogens (Badrhadad et al., 2018). Furthermore, expression of recombinant DrsB1 peptide fused to rice chitinase CBD in tobacco hairy roots and transgenic tobacco plants resulted in higher resistance to plant pathogens (Nazari et al., 2018).

Results of this study revealed that the expression of recombinant peptides in tobacco plants resulted in resistance transgenic plants delaying in disease progression and increased survival under our experimental conditions. This can be possibly explained by employing CBD from a Avr4 effector protein. It is known that upon invasion, *C. fulvum* Avr4 effector secreted from fungal cells competes with plant chitinases for binding to the fungal cell walls chitin through CBD. It has been documented that the chitin binding sites of plant chitinase CBD and race specific Avr4 effector CBD differ in topological and substrate specificity (Jashni et al., 2015). The chitin binding site of Avr4 CBD is larger than plant chitinases CBDs, hence Avr4 binds more strongly to the chitin polymer. Therefore, it can be expected that Avr4 CBD fused to a DrsB1 may more effectively coat the fungal cell wall chitin and brings DrsB1 to the ultimate contacts, eventually leading to better activity of DrsB1 (Van den Burg et al., 2004; Van den Burg et al., 2006). Nevertheless, the expression of an Avr4 CBD from *C. fulvum*, may be considered as an indication of fungal attack and eventually induces the hypersensitivity reactions and overexpression of endochitinases. This phenomenon was observed when *Avr*9 or *Avr*4 genes were transferred to tomato leaves, eliciting a hypersensitive response in transgenic plants (Van der Hoorn et al., 2000). Nonetheless, the later should be investigated to see whether major genes involved in fungal resistance signal transduction are turned on in transgenic plants. The property of Avr4 is that although it is sensitive to host proteases by losing two of the four disulfide bonds in its structure, it retains property of binding to the fungal cell wall chitin and protecting it against plant chitinases (Karimi Jashni et al., 2015). This may suggest that fusion of DrsB1 to CBD not only protect DrsB1 from host degradation but its affinity for fungal cell wall chitin remains unchanged.

Tandem-repeat of carbohydrate-binding domains often cooperates synergistically, resulting in enhancement of the affinity of CBDs for ligand binding (Guillen et al., 2007; Nazarian-Firouzabadi et al., 2007; Nazarian-Firouzabadi et al., 2012). Furthermore, based on our previous studies, the orientation of CBDs to DrsB1 matters. It was anticipated that the (CBD)2-DrsB1 recombinant peptide may have a higher affinity for fungal cell wall chitin, eventually accumulate more DrsB1 peptide to cover fungal plasma membrane in a carpet mode of action (Shai, 2002).

The MIC of the recombination peptides varied for different fungi. A higher concentration of DrsB1 was required to completely inhibit the growth of *A. alternata* and *A. solani* conidia compare with CBD-DrsB1, (CBD)2-DrsB1, and DrsB1-CBD recombinant proteins (**Table 1**), suggesting that recombinant peptides have a higher affinity for fungal cell wall. Interestingly, it has been documented that upon tomato infection, *F. oxysporum* Avr4 effector protein competes with plant chitinases for binding to the fungal cell wall chitin (Jashni et al., 2015). Therefore, it is likely that the fusion of *C. fulvum* Avr4 CBD to DrsB1 peptide outcompetes the fungal pathogens Avr effector proteins and increases the affinity of the recombinant protein toward the fungal cell wall chitin.

It was previously shown that our recombinant peptides had strong antifungal activity *in vitro*, inhibiting the growth of mycelia as well as the germination of conidia and spores at non-toxic concentrations of recombinant peptides isolated from transgenic plants (Khademi et al., 2019; Shams et al., 2019). Regardless of the type of transgene integrated in plants, transgenic lines were more resistant to *Fusarium* species than *Pythium* species. Transgenic lines were able to cope with *Fusarium* infection for more than 2 months, whereas *Pythium* attack led to severe disease in both transgenic and non-transgenic plants in the same period. It has been well-documented that the inhibitory effect of chitinase on the pathogenic fungi depends directly on the proportion of fungi cell wall chitin (Yan et al., 2008), suggesting that the amount of chitin in the fungi cell wall determine the susceptibility of pathogenic fungi to recombinant peptides. The results of this study confirmed that recombinant peptides had a lower activity against *Pythium* fungi with the lowest proportion of cell wall chitin (Yan et al., 2008).

Many cationic peptides are naturally produced by plants as part of their innate defense against pathogenic microorganisms. Previous reports seem contradictory on native AMPs expression as well as degradation by host proteases (Jaynes et al., 1993; Hightower et al., 1994; Huang et al., 1997; Marcos et al., 2008). To address this issue, our study have used pathogen related protein fusions to counter attach microbial invasions. Because our approach harness a natural AMP fused to a part of Avr4 effector protein, there seems to be i) no threat/toxicity to human health and host plant, ii) a reduced chance of developing AMP insensitivity in the pathogen, and iii) a higher resistance against pathogenic fungi, especially fungi with a higher proportion of chitin in their cell wall and iv) a lower chance of host degradation. We think our approach could possibly attract the plant molecular biologist to develop different transgenic disease resistance plants.

## Data Availability Statement

All datasets generated for this study are included in the article/**Supplementary Material**.

## Ethics Statement

The studies involving human participants were reviewed and approved by the Ethics Committee for Research (ECRA) of Lorestan University. Written informed consent was not required according to local and national guidelines.

## Author Contributions

MV-S and MK conducted the experiments. FN-F conceived and designed the research and wrote the manuscript. AI helped in designing some experiments. All authors contributed to the article and approved the submitted version.

## Funding

This research was partly funded by Lorestan University.

## Conflict of Interest

The authors declare that the research was conducted in the absence of any commercial or financial relationships that could be construed as a potential conflict of interest.
