# Supplementary material for: New Recombinant Antimicrobial Peptides Confer Resistance to Fungal Pathogens in Tobacco Plants
Source: Front Plant Sci. 2020 Aug 13;11:1236. doi: 10.3389/fpls.2020.01236 (PMC7438598; doi:10.3389/fpls.2020.01236)
Supplement: Supplementary file 1 [file DataSheet_1.docx]

Supplementary Figure S1

Abbreviations, names and amino acid sequences of different elements of three expression vectors used for tobacco transformation.

1. pGSA/DrsB1-CBD:

SP+His+DrsB1+L2+CBD

1. pGSA/CBD-DrsB1:

SP+His+CBD+L2+DrsB1

1. pGSA/(CBD)2-DrsB1:

SP+His+CBD+L1+CBD+L2+DrsB1

| Amino acid sequences | Name | Expression vector elements |
| --- | --- | --- |
| MAHYTTLLLSTLLVGTALAQ | Avr4 signal peptide | SP |
| RGSHHHHHH | His-tag | His |
| AMWKDVLKKIGTVALHAGKAALGAVADTISQ | DrsB1: Dermaseptin B1 | DrsB1 |
| TTTRAPTTTTKSAPTVTTTTRAPTTTTPA | Rice chitinase helix-forming linker (EAAAK)4 (GenBank accession No.: X54367.1) | L1 |
| EAAAKEAAAKEAAAKEAAAK | Linker sequence from *Caenorhabditis elegans* chitinase (CCD73759) gene | L2 |
| TKCMGPKDCLYPNPDSCTTYIQCVPLDEVGNAKPVVKPCPKGLQWNDNVGKKWCDYPNLSTCPV | Carbohydrate binding domain from Avr4 | CBD |
