# Supplementary material for: New Recombinant Antimicrobial Peptides Confer Resistance to Fungal Pathogens in Tobacco Plants
Source: Front Plant Sci. 2020 Aug 13;11:1236. doi: 10.3389/fpls.2020.01236 (PMC7438598; doi:10.3389/fpls.2020.01236)
Supplement: Supplementary file 2 [file DataSheet_2.docx]

**Supplementary Figure S2.** The diagram showing the hemolysis activity of recombinant peptides on human erythrocytes.
