# Supplementary material for: New Recombinant Antimicrobial Peptides Confer Resistance to Fungal Pathogens in Tobacco Plants
Source: Front Plant Sci. 2020 Aug 13;11:1236. doi: 10.3389/fpls.2020.01236 (PMC7438598; doi:10.3389/fpls.2020.01236)
Supplement: Supplementary file 3 [file Table_1.docx]

Supplementary Table 1: ANOVA table showing the inhibitory effects of the recombinant peptides on growth of the six fungal species

| Mean squares | df | Source of variation |
| --- | --- | --- |
| 4015.40^**^ | 5 | Fungi |
| 1313.70^**^ | 6 | Lines |
| 117.48^**^ | 30 | Fungi× Lines |
| 1.65 | 84 | Error |
| 5.74% |  | CV (%) |

**Significant at P<0.01
