# Supplementary material for: New Recombinant Antimicrobial Peptides Confer Resistance to Fungal Pathogens in Tobacco Plants
Source: Front Plant Sci. 2020 Aug 13;11:1236. doi: 10.3389/fpls.2020.01236 (PMC7438598; doi:10.3389/fpls.2020.01236)
Supplement: Supplementary file 4 [file Table_2.docx]

| **Supplementary Table S2.** Analysis of variance of Disease Severity on detached leaves of transgenic and control plants against fungal disease. The diagram showing the disease severity of the detached leaves. The disease severity is calculated based on the ratio of the damaged area to the total leaf area. The antifungal activity data were statistically analyzed as a completely randomized design for each fungus and the mean comparison was done by Least significant differences (LSD) test in three replicates using SAS 9.1 (SAS, Inc., North Carolina, USA) software. The p value of ≤0.01 was considered as significant. | | | |  |
| --- | --- | --- | --- | --- |
| Mean squares | df | Source of variation |  |  |
| Disease Severity (% leaf area) |  |  | Fungal |  |
| 307.83** | 6 | lines | *A. alternata* |  |
| 6.14 | 14 | Error |  |  |
| 13.8 |  | CV |  |  |
| 336.00** | 6 | lines | *A. solani* |  |
| 112.00 | 14 | Error |  |  |
| 12.8 |  | CV |  |  |
| 702.65** | 6 | lines | *F. oxysporum* |  |
| 12.24 | 14 | Error |  |  |
| 10.5 |  | CV |  |  |
| 702.00** | 6 | lines | *F. solani* |  |
| 10.71 | 14 | Error |  |  |
| 8.6 |  | CV |  |  |
| 407.65** | 6 | lines | *Pythium* sp. |  |
| 6.67 | 14 | Error |  |  |
| 3.8 |  | CV |  |  |
| 2.63^n.s^ | 6 | lines | *P. aphanydermatum* |  |
| 3.57 | 14 | Error |  |  |
| 1.96 |  | CV |  |  |
| ** Significant at P ≤0.01; ns: non-significant at P≥0.05; CV: Coefficient of variation. | | | | |
