# Supplementary material for: New Recombinant Antimicrobial Peptides Confer Resistance to Fungal Pathogens in Tobacco Plants
Source: Front Plant Sci. 2020 Aug 13;11:1236. doi: 10.3389/fpls.2020.01236 (PMC7438598; doi:10.3389/fpls.2020.01236)
Supplement: Supplementary file 5 [file Table_3.docx]

Supplementary Table S3 . Analysis of variance for growth characteristics of transgenic and control plants challenged with fungal pathogens.

| Mean squares | | | | | | df | Source of variation |
| --- | --- | --- | --- | --- | --- | --- | --- |
| Root dry  weight | Shoot dry  weight | Root fresh  weight | Stem fresh weight | Root  height | Shoot  height |  |  |
| 3.76** | 16.86** | 434.03** | 706.10** | 643.10** | 611.05** | 2 | Fungi |
| 1.80** | 3.10** | 143.82** | 87.64** | 121.95** | 166.34** | 6 | lines |
| 0.04** | 0.29** | 5.69** | 3.53** | 4.70** | 5.92** | 12 | Fungi× lines |
| 0.001 | 0.001 | 0.02 | 0.18 | 0.13 | 0.05 | 42 | Error |
| 1.67 | 0.70 | 0.77 | 1.37 | 1.15 | 0.53 |  | CV |

** Significant at α=0.01 probability level. CV: Coefficient of variation
